# Supplementary material for: Immune Response and Breakthrough Infection Risk After SARS-CoV-2 Vaccines in Patients with Hemoglobinopathy: A Single Center Experience
Source: Vaccines (Basel). 2025 Jan 23;13(2):111. doi: 10.3390/vaccines13020111 (PMC11860232; doi:10.3390/vaccines13020111)
Supplement: Supplementary file 1 [file vaccines-13-00111-s001.zip › vaccines-3346393-supplementary.pdf]

SUPPLEMENTARY FILES

Table S1. Raw data of 114 enrolled patients, as described in the text.

PATIENT'S CODE	AGE	IgG ANTI N	RESULT	IgG ANTI S	BAU	RESULT
HB 21/023	45	0.02 Index	Negative	11419.3	**1621,5406**	Positive
HB 21/038	52	0.03 Index	Negative	40000	**5680**	Positive
HB 21/154	48	0.08 Index	Negative	392.8	**55,7776**	Positive
HB 21/039	50	0.01 Index	Negative	3505.4	**497,7668**	Positive
HB 21/040	53	0.04 Index	Negative	3250,5	**461,571**	Positive
HB 21/083	51	0.08 Index	Negative	18028,9	**2560,1038**	Positive
HB 21/076	70	0.02 Index	Negative	18787.1	**2667,7682**	Positive
HB 21/145	81	0.03 Index	Negative	7890.3	**1120,4226**	Positive
HB 21/011	29	0.02 Index	Negative	29258.4	**4154,6928**	Positive
HB 21/084	25	0.02 Index	Negative	14484.0	**2056,728**	Positive
HB 21/141	25	0.02 Index	Negative	1998.2	**283,7444**	Positive
HB 21/053	27	0.16 Index	Negative	36177.8	**5137,2476**	Positive
HB 21/063	36	0.02 Index	Negative	8121,5	**1153,253**	Positive
HB 21/054	48	0.03 Index	Negative	16271.4	**2310,5388**	Positive
HB 21/136	55	0.07 Index	Negative	6764.1	**960,5022**	Positive
HB 21/041	40	0.01 Index	Negative	23014.6	**3268,0732**	Positive
HB 21/064	32	0.17 Index	Negative	11098.5	**1575,987**	Positive
HB 21/024	49	0.79 Index	Negative	9596.2	**1362,6604**	Positive
HB 21/137	50	0.45 Index	Negative	2286.9	**324,7398**	Positive
HB 21/089	51	0.80 Index	Negative	17731.2	**2517,8304**	Positive
HB 21/077	50	0.01 Index	Negative	21300.3	**3024,6426**	Positive
HB 21/133	46	0.08 Index	Negative	21553.8	**3060,6396**	Positive
HB 21/078	67	0.02 Index	Negative	40000	**5680**	Positive
HB 21/090	75	0.47 Index	Negative	5347.5	**759,345**	Positive
HB 21/119	37	0.08 Index	Negative	9824,9	**1395,1358**	Positive
HB 21/055	34	0.18 Index	Negative	40000	**5680**	Positive
HB 21/091	50	0.58 Index	Negative	2029.9	**288,2458**	Positive
HB 21/001	45	0.05 Index	Negative	40000	**5680**	Positive
HB 21/065	75	0.03 Index	Negative	22250.1	**3159,5142**	Positive
HB 21/042	58	0.01 Index	Negative	15514.5	**2203,059**	Positive
HB 21/085	41	0.19 Index	Negative	12843.4	**1823,7628**	Positive
HB 21/079	55	0.09 Index	Negative	33345.0	**4734,99**	Positive

HB 21/127	22	0.06 Index	Negative	36813.0	**5227,446**	Positive
HB 21/043	80	0.09 Index	Negative	204.0	**28,968**	Positive
HB 21/086	65	0.04 Index	Negative	5553.7	**788,6254**	Positive
HB 21/013	48	0.02 Index	Negative	40000	**5680**	Positive
HB 21/014	45	0.04 Index	Negative	12052.5	**1711,455**	Positive
HB 21/080	45	0.04 Index	Negative	8413.2	**1194,6744**	Positive
HB 21/015	46	0.02 Index	Negative	15384.8	**2184,6416**	Positive
HB 21/016	34	0.40 Index	Negative	16638.3	**2362,6386**	Positive
HB 21/122	41	0.03 Index	Negative	6818.3	**968,1986**	Positive
HB 21/027	44	0.04 Index	Negative	16064.6	**2281,1732**	Positive
HB 21/060	56	0.04 Index	Negative	24838.4	**3527,0528**	Positive
HB 21/056	49	0.03 Index	Negative	29523.4	**4192,3228**	Positive
HB 21/092	52	0.03 Index	Negative	13984.9	**1985,8558**	Positive
HB 21/101	55	0.01 Index	Negative	40000	**5680**	Positive
HB 21/066	48	0.06 Index	Negative	12578.2	**1786,1044**	Positive
HB 21/067	28	0.01 Index	Negative	40000	**5680**	Positive
HB 21/120	24	0.02 Index	Negative	25821.9	**3666,7098**	Positive
HB 21/116	47	0.02 Index	Negative	25809.4	**3664,9348**	Positive
HB 21/081	50	0.03 Index	Negative	8258.9	**1172,7638**	Positive
HB 21/002	46	0.05 Index	Negative	16625.2	**2360,7784**	Positive
HB 21/003	56	0.03 Index	Negative	40000	**5680**	Positive
HB 21/004	58	0.03 Index	Negative	40000	**5680**	Positive
HB 21/114	42	0.03 Index	Negative	12124	**1721,608**	Positive
HB 21/028	40	0.78 Index	Negative	7994.3	**1135,1906**	Positive
HB 21/068	71	0.04 Index	Negative	11952,3	**1697,2266**	Positive
HB 21/125	29	0.02 Index	Negative	340.9	**48,4078**	Positive
HB 21/045	45	0.02 Index	Negative	31886.4	**4527,8688**	Positive
HB 21/093	35	0.02 Index	Negative	3200,2	**454,4284**	Positive
HB 21/069	46	0.14 Index	Negative	36784,3	**5223,3706**	Positive
HB 21/005	38	1.69 Index	Positive	40000	**5680**	Positive
HB 21/139	51	0.02 Index	Negative	23976.7	**3404,6914**	Positive
HB 21/046	54	0.03 Index	Negative	3153.4	**447,7828**	Positive
HB 21/128	74	0.02 Index	Negative	9343.4	**1326,7628**	Positive
HB 21/047	50	0.04 Index	Negative	3595.0	**510,49**	Positive
HB 21/143	65	0.17 Index	Negative	7539.3	**1070,5806**	Positive
HB 21/006	42	0.05 Index	Negative	18564.6	**2636,1732**	Positive
HB 21/070	59	0.05 Index	Negative	19471,8	**2764,9956**	Positive
HB 21/115	46	0.24 Index	Negative	36490.3	**5181,6226**	Positive

HB 21/029	39	0.03 Index	Negative	19959.7	**2834,2774**	Positive
HB 21/129	82	0.04 Index	Negative	38494.9	**5466,2758**	Positive
HB 21/018	37	0.18 Index	Negative	36555.4	**5190,8668**	Positive
HB 21/071	47	0.03 Index	Negative	27446.5	**3897,403**	Positive
HB 21/123	50	0.19 Index	Negative	25693.3	**3648,4486**	Positive
HB 21/061	40	0.03 Index	Negative	6211,8	**882,0756**	Positive
HB 21/072	24	0.04 Index	Negative	11881.6	**1687,1872**	Positive
HB 21/094	49	0.03 Index	Negative	15244,7	**2164,7474**	Positive
HB 21/057	46	0.03 Index	Negative	22022.7	**3127,2234**	Positive
HB 21/062	34	0.28 Index	Negative	33923.1	**4817,0802**	Positive
HB 21/019	44	0.09 Index	Negative	7921.3	**1124,8246**	Positive
HB 21/020	71	0.14 Index	Negative	18634.4	**2646,0848**	Positive
HB 21/007	54	0.78 Index	Negative	10421.4	**1479,8388**	Positive
HB 21/048	43	0.05 Index	Negative	28539.8	**4052,6516**	Positive
HB 21/032	57	0.07 Index	Negative	27983.9	**3973,7138**	Positive
HB 21/073	31	0.03 Index	Negative	40000	**5680**	Positive
HB 21/033	69	0.03 Index	Negative	40000	**5680**	Positive
HB 21/082	25	0.02 Index	Negative	17475.8	**2481,5636**	Positive
HB 21/142	51	0.66 Index	Negative	3702.0	**525,684**	Positive
HB 21/008	46	0.09 Index	Negative	40000	**5680**	Positive
HB 21/009	59	0.07 Index	Negative	4392.0	**623,664**	Positive
HB 21/095	53	0.72 Index	Negative	40000	**5680**	Positive
HB 21/087	40	0.04 Index	Negative	7278.4	**1033,5328**	Positive
HB 21/134	52	0.02 Index	Negative	3912.4	**555,5608**	Positive
HB 21/117	62	0.02 Index	Negative	40000	**5680**	Positive
HB 21/088	45	0.08 Index	Negative	40000	**5680**	Positive
HB 21/050	46	0.39 Index	Negative	28662.4	**4070,0608**	Positive
HB 21/124	56	0.02 Index	Negative	12832.0	**1822,144**	Positive
HB 21/051	48	0.02 Index	Negative	15355.6	**2180,4952**	Positive
HB 21/121	25	0.02 Index	Negative	40000	**5680**	Positive
HB 21/035	32	0.02 Index	Negative	24158.8	**3430,5496**	Positive
HB 21/140	78	0.05 Index	Negative	15674.8	**2225,8216**	Positive
HB 21/036	41	0.19 Index	Negative	8533.9	**1211,8138**	Positive
HB 21/010	32	0.29 Index	Negative	40000	**5680**	Positive
HB 21/135	40	0.02 Index	Negative	31377.0	**4455,534**	Positive
HB 21/074	27	0.03 Index	Negative	15381.9	**2184,2298**	Positive
HB 21/021	37	0.05 Index	Negative	16546.1	**2349,5462**	Positive

HB 21/022	42	0.07 Index	Negative	32929.6	**4676,0032**	Positive
HB 21/037	50	0.21 Index	Negative	14844.0	**2107,848**	Positive
HB 21/118	31	0.10 Index	Negative	35973.2	**5108,1944**	Positive
HB 21/059	32	0.14 Index	Negative	12450.1	**1767,9142**	Positive
HB 21/126	29	0.06 Index	Negative	6230.9	**884,7878**	Positive
HB 21/075	52	0.02 Index	Negative	1554.6	**220,7532**	Positive
HB 21/144	32	0.03 Index	Negative	29254.3	**4154,1106**	Positive
